# The under-representation of racially minoritised doctors in academic general practice training: a retrospective analysis

**DOI:** 10.3399/BJGPO.2023.0136

**Published:** 2024-04-17

**Authors:** Alice Howe, Chloe Orkin, Vanessa Apea

**Affiliations:** 1 SHARE Collaborative, Wolfson Institute of Population Health, Queen Mary University of London, London, UK; 2 Tower Hamlets Primary Care Trust, London, UK; 3 Barts Health NHS Trust, London, UK; 4 SHARE Collaborative, Blizard Institute, Queen Mary University of London, London, UK

**Keywords:** ethnicity, inclusion, academia, general practice

## Abstract

**Background:**

General practice has one of the most diverse medical training programmes in terms of sex and ethnic background. However, this diversity of race and ethnicity is not reflected in academic GP careers, with just 17% (*n* = 81/473) of academic GPs being from racially minoritised groups, according to the Medical Schools Council.

**Aim:**

To determine whether GP academic clinical fellow (ACF) trainees from racially minoritised backgrounds are proportionally represented, compared with the non-academic training programme, using the annual GP ACF conference as a proxy.

**Design & setting:**

A retrospective analysis of conference programmes from national academic GP training conferences from 2018–2023 and demographic data obtained from Health Education England (HEE).

**Method:**

Using conference programmes and online searches, demographic information on conference speakers was obtained and a freedom of information request was made to HEE for the demographics of GP ACFs for corresponding years. This was compared with demographic data of GP trainees and academics.

**Results:**

On average, there were 40 speakers each year at the conference. White females (average 20.2 speakers each year) were the most well represented group, followed by White males (average 12.5), Asian females (average 3.3), Asian males (average 1.8), Black males (average 0.7), and Black females (average 0.3). HEE data from 2022 revealed that 27 (71.1%) of the 38 (excluding five who did not state their ethnicity) ACFs were White British.

**Conclusion:**

GP academia should be more representative of the non-academic GP training scheme. Work needs to be done to understand and overcome the structural barriers to recruiting from racially minoritised groups.

## How this fits in

The Medical Workforce Race Equality Standard (MWRES) 2022 highlighted that doctors from so-called ‘Black and Minority Ethnic (BME)’ backgrounds have a worse experience than doctors from White backgrounds (in terms of harassment, bullying, and abuse from other staff members), are paid less, and shortlisted for and offered proportionally fewer consultant jobs than their White counterparts. However, the MWRES does not yet include GPs and little is known about the experience of doctors from racially minoritised backgrounds about academic career progression within general practice.

## Introduction

The NHS workforce is more diverse than ever, with 24.2% of the overall workforce being from racially minoritised backgrounds.^1^ Many resources now exist, such as the Workforce Race Equality Standard (WRES) and the Medical Workforce Race Equality Standard, to highlight and document the disparities in the experiences and progression of racially minoritised staff members within the NHS. The General Medical Council (GMC) and the Medical Schools Council (MSC) also collect demographic data on so-called ‘Black, Asian and Minority Ethnic’ (BAME) groups to highlight these differences. This study aimed to add to this knowledge, in the specific area of general practice.

While we value the data from the aforementioned sources, the authors of this study do not align with the terminology of BME or BAME that is often used by these organisations. We prefer the term racially minoritised as it reflects the minoritisation of individuals through social processes of power. However, we use ‘so-called BAME or BME’ throughout this study when directly quoting data from these organisations in order to reflect their data accurately.

In England and Wales, 81.7% of the population are White, 4.0% are Black, 9.3% are Asian, 2.9% are of mixed ethnicity, and 2.1% are classed as ‘other ethnicity’, according to the 2021 consensus.^2^ Comparatively, doctors in England, in 2022, had a much higher proportional representation of racially minoritised individuals, with 49.9% of the medical workforce coming from racially minoritised groups (after excluding unknowns).^3^ For medical trainees this proportion is even higher at 55.0%, with the highest proportion seen in the non-consultant specialist training group (65% after excluding unknowns).^1^

With respect to GPs specifically, the representation of racially minoritised doctors is in itself high. In August 2023, out of a total of 46 853 GPs and GP trainees in England, 22 675 (48.4%) were White, 12 075 (25.8%) were Asian, 2920 (6.2%) were Black, 1135 (2.4%) were of mixed race and/or ethnicity, 1775 (3.8%) were from ‘other’ ethnic group, and 6800 (14.5%) were unknown ethnicity or not stated.^4^ For comparison, excluding unknowns, 44.1% of the GP workforce in England is made up of those from racially minoritised groups.

Nationally, the GMC has reported that <50% of GP trainees were White.^5^ The only other specialties with <50% White trainees were ophthalmology and radiology.^5^ This is largely owing to a 239% increase in international medical graduates (IMGs) to the GP register,^5^ which has also contributed to the proportion of Black or Black British trainees in general practice trebling from 5% in 2012 to 15% in 2021.^5^ In August 2023, out of a total of 10 446 GP trainees in England, 5379 (51.5%) were from the UK, 594 (5.7%) were from the EU, 4462 (42.7%) were from outside of the UK or EU, and 11 (0.1%) were from an unknown location.^4^ Work by Beckwith *et al* ^6^ found that postgraduate academic trainees were significantly more likely to be White but found no significant difference in the ethnicities of UK graduates in academic posts, as opposed to internationally trained graduates. This suggests that the intersections of country of origin, race, and ethnicity may compound inequitable recruitment.

With this degree of diverse representation in the GP medical workforce, it would be plausible to predict this would translate into similar proportions of those from a racially minoritised background in academic, senior, and leadership roles within general practice. However, this is not the case and suggests inequity of opportunity.

The MSC^7^ collects data regarding clinical academics in the UK. According to MSC, there were 500 academic GPs in the UK in 2022, including professors (*n* = 87, 17.4%), reader or senior lecturers (*n* = 213, 42.6%), and lecturers (*n* = 200, 40.0%). By race and ethnicity, 73 (83.9%) of the professors were White, 10 (11.5%) were from so-called BME backgrounds, and the race and ethnicity of the other four (4.6%) was unknown. Of the reader or senior lecturers, 165 (77.5%) were White, 39 (18.3%) were from so-called BME backgrounds, and nine (4.2%) were unknown race and/or ethnicity. For lecturers, 139 (69.5%) were White, 46 (23.0%) were from so-called BME backgrounds, and 15 (7.5%) unknown. When looking specifically at the disaggregated data of full-time equivalent clinical academic GPs, within the so-called BME category, there are zero (to the nearest 5) Black, Chinese or mixed ethnicity professors, senior lecturers, or lecturers.^7^ By sex there were 54 (62.1%) male professors in general practice and 33 (37.9%) females. There were 83 (39.0%) males in reader or senior lecturer roles in general practice and 130 (59.9%) females. There were 73 (36.5%) male lecturers and 127 (63.5%) female lecturers. For comparison, 57% of the overall GP workforce is female.^4^ This demonstrates a sex attainment gap with respect to seniority.

The National Institute for Health and Care Research (NIHR) Academic Clinical Fellowship training programme is aimed at recruiting junior doctors with both interest in and potential to become clinical academics. Fair and representational recruitment to the programme directly affects the future academic pipeline as the role is often seen as the gateway to a clinical academic career. There are 43 NIHR GP academic clinical fellow (ACF) training posts each year in the UK. Annually, a national academic GP training conference provides the opportunity for GP ACFs to present their work, share ideas, and network with other academic GPs. Presenting at conferences is regarded as necessary for career progression within academia and as a good way to identify collaboration opportunities.

The aim of this study was to determine whether GP ACF trainees from racially minoritised backgrounds are proportionally represented in the annual GP ACF conferences and, furthermore, if doctors from racially minoritised backgrounds are proportionally represented in GP clinical academia compared with the non-academic GP training scheme. The objectives were to utilise the demographics of speakers at the conference as a proxy for representation at a junior level and then align this with a freedom of information (FoI) request from Health Education England (HEE). Then to compare how doctors from racially minoritised backgrounds are represented in clinical academia.

## Method

A retrospective analysis of the last six programmes, from 2018–2023, from the national academic GP training conferences, which are freely available online, was conducted to determine the names and biographies of speakers. The researcher (AH) then used online search engines to capture online profiles and information about the speaker. A code was assigned to the person whether they appeared to be a White female, White male, Asian female, Asian male, Black female, or Black male. A different code was also assigned if no information could be found on the speaker. The conferences were held in Oxford, Manchester, Bristol, London, Cambridge, and Edge Hill, chronologically.

A FoI request was submitted to HEE, asking for the demographic information of the 43 ACF positions across the UK for five of the corresponding years: 2018–2022 (appointments for 2023 had not been made at the time of the request).

The MSC clinical academic survey was reviewed to find out the demographic information of the clinical academic GP population, in order to compare it with the demographic data of non-academic trainees and ACF trainees. The MSC is the representative body for the UK medical schools.

## Results

**Figure 1.**
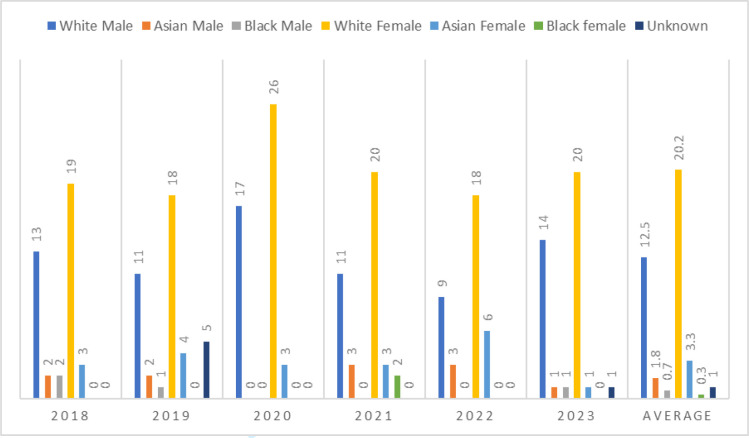
Graphical representation of number of speakers, by sex and race and ethnicity at annual GP academic clinical fellow conferences 2018–2023

On average, there were 40 speakers each year at the conference. White females (average 20.2 speakers each year) were the most well represented group, followed by White males (average 12.5), Asian females (average 3.3), Asian males (average 1.8), Black males (average 0.7), and Black females (average 0.3). The trend was consistent every year with representation of each group remaining largely unchanged throughout the 6 years (see Figure 1 and Table 1).

**Table 1.** Number and percentage of speakers at GP academic clinical fellow conferences from 2018–2023, by sex and race and ethnicity. Data presented as *n* (%)

Speaker ethnicity and sex	**2018**	**2019**	**2020**	**2021**	**2022**	**2023**	Average
White male	13 (33.3)	11 (26.8)	17 (37.0)	11 (28.2)	9 (25.0)	14 (36.8)	12.5 (31.4)
Asian male	2 (5.1)	2 (4.9)	0 (0)	3 (7.7)	3 (8.3)	1 (2.6)	1.8 (4.5)
Black male	2 (5.1)	1 (2.4)	0 (0)	0 (0)	0 (0)	1 (2.6)	0.7 (1.8)
White female	19 (48.7)	18 (43.9)	26 (56.5)	20 (51.3)	18 (50.0)	20 (52.6)	20.2 (50.8)
Asian female	3 (7.7)	4 (9.8)	3 (6.5)	3 (7.7)	6 (16.7)	1 (2.6)	3.3 (8.3)
Black female	0 (0)	0 (0)	0 (0)	2 (5.1)	0 (0)	0 (0)	0.3 (0.8)
Unknown	0 (0)	5 (12.2)	0 (0)	0 (0)	0 (0)	1 (2.6)	1.0 (2.5)
Total	39	41	46	39	36	38	**39.8**

In November 2022, a FoI request from HEE provided data on the two most recent intakes (2021 and 2022). Owing to HEE’s retention policy, only 2 years of information were provided. In 2021, only three people, out of a total of 43, declared their race and ethnicity and they were all White (White British or other White background). Four declared their sex in 2021 and the numbers were too small to know how many were male or female.

In 2022, 38 (88.4%) of the total 43 people who accepted ACF jobs declared their ethnicity. Of these 38, 27 (71.1%) were White British (62.8% of the total number of ACF jobs). The remaining 11 were from minority ethnic groups, spread over seven ethnic groups, as shown in Table 2, which details the ethnicity and sex of trainees who accepted ACF jobs. Numbers were too small to differentiate the exact numbers of each racially minoritised group but <5 were Black or Black British Caribbean, <5 were from ‘other ethnic group’, and the remainder were from Asian ethnic groups. The sex split in 2022 was 19 (44.2%) male, 20 (46.5%) female, and four (9.3%) unknown.

**Table 2.** Ethnic group and sex of trainees who accepted ACF GP jobs in 2021 and 2022 (where applicable, rounded to the nearest 5). Data presented as *n* (%). Data from Health Education England.

Characteristic	2021	2022
**Ethnic group**		
White British	<5 (<11.6)	27 (62.8)
White Irish	0 (0)	0 (0)
Any other White background	<5 (<11.6)	0 (0)
Mixed White and Black Caribbean	0 (0)	0 (0)
Mixed White and Black African	0 (0)	0 (0)
Mixed White and Asian	0 (0)	<5 (<11.6)
Any other mixed background	0 (0)	0 (0)
Asian or Asian British Indian	0 (0)	<5 (<11.6)
Asian or Asian British Pakistani	0 (0)	<5 (<11.6)
Asian or Asian British Bangladeshi	0 (0)	<5 (<11.6)
Any other Asian background	0 (0)	0 (0)
Black or Black British Caribbean	0 (0)	<5 (<11.6)
Black or Black British African	0 (0)	0 (0)
Any other Black background	0 (0)	0 (0)
Chinese	0 (0)	<5 (<11.6)
Any other ethnic group	0 (0)	<5 (<11.6)
Not stated	40 (93.0)	5 (11.6)
**Sex**		
Male	<5 (<11.6)	19 (44.2)
Female	<5 (<11.6)	20 (46.5)
Not stated	39 (90.7)	4 (9.3)
**Total**	**43**	**43**

When comparing non-academic GP trainees with academic GP trainees, there are proportionally fewer trainees from racially minoritised groups in academic posts, compared with non-academic posts (Table 3).

**Table 3.** Table comparing non-academic GP trainees with academic GP trainees and clinical academics

	GP or GP trainees, %^2,4^	GP ACFs (UK 2022) HEE, %	GP clinical academics, %^7^
White	48.4	62.8	75.4
Asian	25.8	<18.6	19.0
Black	6.0	<11.6	
Mixed	2.4	<11.6	
Other	3.8	<11.6	
Unknown	14.5	11.6	5.6

ACF = academic clinical fellows. HEE = Health Education England

## Discussion

### Summary

Based on the ACF intake levels in 2022, of whom, 71.1% were White British (of the 38 people who disclosed their ethnicity), one would expect a similar proportion of White speakers at the annual GP ACF conference. However, over a period of 6 years, on average, 82% of the speakers were White, which is disproportionately high. This would imply that the small proportion of doctors from racially minoritised backgrounds who are in ACF posts do not participate as a speaker at the national ACF conference; either by choice or lack of opportunity.

This work highlights clear racial and ethnic disparities in attainment within GP academia; extending from recruitment into GP ACF posts and continuing across the entire GP clinical academic pathway. Racially minoritised doctors become proportionally less well represented as the academic ladder increases in seniority, evidencing an ethnicity attainment gap. This reflects the 'leaky pipeline’ phenomenon, where there is a progressive loss of capable persons from academic clinical careers. This is particularly evident in Black groups in general practice. In 2021, 15% of the UK GP training cohort were Black but just 6% of all GP and trainee GPs in England were Black. Also, <1% of clinical lecturers, senior lecturers and professors in general practice were Black, with numbers too small to be more accurate. Even at ACF level, <2% of the 2022 cohort were Black.

Possible reasons for doctors from racially minoritised backgrounds not entering into academia include not being aware of these roles, being deterred from these roles, not having visible representation in role models or mentors, and perceived or experiential institutional racism. This starts in medical school, where students from racially minoritised backgrounds often feel that there is a lack of role modelling and mentoring.^8^ Early career researchers from racially minoritised groups have been noted to be disadvantaged owing to patronisation and paternalism, lack of funding, and the ethnicity pay gap.^9–11^ The losses multiply at each level culminating in capable doctors and potential academics from racially minoritised backgrounds being lost.

It is important to promote GP academia as an attractive career choice. A survey of medical students found that only 3% thought that a career in general practice would be intellectually challenging and 39% reported that tutors from other specialties had put them off a career in general practice.^12^ In 2016, the Wass report^13^ made recommendations to raise the profile and improve the vision of general practice. This included a specific recommendation that academic training opportunities were properly supported and promoted. However, Lawson and Kumar specify five areas in which we are still failing, including widening access in medical schools and role modelling in general practice,^14^ both of which could be relevant to removing barriers for doctors from racially minoritised backgrounds and IMG trainees.

### Strengths and limitations

Limitations of our method include race and ethnicity assigned to those included in the analysis was not self-reported but rather assigned by the researcher, based on their appearance and their online profile. We recognise that this might not always be the same race and ethnicity that the speaker might assign for themselves. We used three broad race and ethnicity categories but we acknowledge that there are far more than this and that by doing this ourselves we are being reductionist, failing to account for the vast heterogeneity of the workforce. As such, we understand that this method will lose granularity and nuanced differences will be missed. However, we believe that this is an important start in highlighting the problem.

### Comparison with existing literature

A recent systematic review and qualitative study exploring the inequalities in UK clinical academic careers by Brown *et al* found that women and doctors from so-called ‘BME’ backgrounds are less likely to progress through clinical academic careers.^9^ Unfortunately, most work in this area comes from the US and work needs to be done within the UK to understand the barriers and facilitators into an academic clinical career. Their study was extensive and emphasised a real inequity in clinical academia in the UK. However, it was broad and it focused more on sex than ethnicity.

Previous research by the authors of this study at Queen Mary University of London assessed the intersectionality of presenters at conferences and analysed how this affected conference participation. The work showed that when there is more diversity on the panel, there is more participation from the audience.^15^ Having better representation of racially minoritised clinical academics at conferences may not only increase participation, but also lead to role modelling within racially minoritised groups.

### Implications for research and practice

Our work shines a spotlight on the ‘glass ceiling’ affecting GPs from racially minoritised backgrounds within academia, especially for Black or Black British GPs entering into or progressing through academia. The WRES 2022 report^1^ shows that so-called BME staff are less likely to think that their trust provides equal opportunities for career progression or promotion (58.7% of White staff compared with 44.4% of staff from so called BME backgrounds). This is even lower for Black staff groups, with only 35.4% believing there are fair opportunities. This is problematic and work needs to be done to explore this lack of opportunity further. However, the WRES report excludes the experiences of GPs and it would be interesting to know if the same patterns apply in primary care. We have highlighted a gap in the research and present on the racial and ethnic disparity at GP ACF level, which, to the authors’ knowledge, has not been done before.

The reasons behind these findings are important and worthy of study. Further work should explore whether ACF trainees from racially minoritised backgrounds are submitting abstracts and getting rejected or if they have not submitted abstracts at all. Qualitative research to better understand the issues may be helpful in exploring themes around lack of role modelling, lack of confidence, micro-aggressions, and institutional racism.

Further work should also include qualitative research on the experiences of GP ACF trainees from a range of racially minoritised backgrounds, early career researchers, and of those who have not been able to pursue a career in academia for whatever reason. This work should also disaggregate and distinguish between different so-called BME groups in order to highlight where further work needs to be done. This will enable us to better understand the barriers and facilitators to an academic clinical career as a GP.

By being complacent, we are being complicit in this injustice. We must call out racist structures, policies, and behaviour and ensure that we contribute to fair and representational recruitment into academic and senior roles. Not only is there a moral imperative, but also diversity in our workforce is good for patients, good for juniors, and good for the wider team. By harnessing the skills, knowledge, and perspectives of GPs from all backgrounds, we can move to providing truly holistic care for our patients. Research tells us that a diverse workforce improves performance, creativity, and can lead to more impactful research.^16^ It can allow patients from similar backgrounds to feel represented as well as providing junior colleagues from racially minoritised backgrounds with role models. We must be active in our pursuit of becoming an anti-racist institution, organisation, or entity. Not being actively racist is not enough.
